# Latinos’ diminished returns of educational attainment on reducing food insecurity: the role of ethnic disparities in family structure and employment

**DOI:** 10.3389/fpubh.2024.1407005

**Published:** 2024-08-19

**Authors:** Shervin Assari

**Affiliations:** Department of Internal Medicine, Charles R Drew University of Medicine and Science, Los Angeles, CA, United States

**Keywords:** minorities’ diminished returns, Latino, non-Latino white, education, health disparities, food security

## Abstract

**Background:**

Higher education is widely recognized as a strategy to mitigate food insecurity. However, marginalized and racialized groups, especially Latinos, often do not experience the same economic and health benefits from their educational achievements as non-Latino Whites, highlighting a pattern of diminished returns within these communities.

**Aims:**

This study aims to explore the disparities in how educational attainment influences marital status and employment, and subsequently, food insecurity among Latino and non-Latino adults.

**Methods:**

Utilizing data from the 2022 National Health Interview Survey (NHIS), which encompassed 27,648 adults from both Latino and non-Latino backgrounds, this research applied a structural equation model to examine the relationship between educational attainment, ethnicity, and food insecurity. The study specifically focused on the mediating roles of marital status and employment.

**Results:**

Findings reveal significant interactions between education and ethnicity affecting marital status and employment, both of which serve as protective factors against food insecurity. These results indicate that higher levels of unemployment and lower marriage rates may disproportionately escalate food insecurity among Latinos, irrespective of educational attainment.

**Conclusion:**

The study highlights profound societal and environmental obstacles that prevent Latinos from leveraging educational achievements to improve their marital and employment statuses, and thereby, their food security. Addressing these disparities demands targeted interventions directed at Latino communities to bridge gaps in employment and marriage rates stemming from educational disparities. A holistic strategy that transcends mere access to education is essential to dismantle the societal barriers that undermine the educational dividends for Latino communities.

## Background

Ensuring universal access to nutritious food is crucial for advancing health equity, particularly for children in marginalized and socioeconomically disadvantaged groups who face risks like malnutrition and poor academic achievement due to food insecurity (1). By acknowledging the universal right to healthy food, it underscores the responsibility of the United States government to guarantee that everyone, regardless of socioeconomic status, has access to affordable and nutritious food (2, 3). This commitment is vital for fostering a more equitable and healthy future (4). Food insecurity is linked to significant health risks, including chronic diseases and mental health issues, and erodes social cohesion by increasing feelings of alienation (5–8). There is a pressing need for comprehensive strategies to ensure access to nutritious food and to address the broad consequences of food insecurity (9, 10).

Food insecurity inflicts significant harm, affecting individuals and communities on various levels (11). It is linked with a greater risk of chronic conditions like obesity, diabetes, and cardiovascular diseases, often due to reliance on less nutritious, cheaper food options (12). The stress and anxiety from food insecurity can also impact mental health, leading to depression, diminished academic performance in children, and decreased productivity in adults (5–8). Furthermore, food insecurity disrupts social cohesion, isolating affected individuals and families from social and cultural food-related practices, exacerbating feelings of alienation and exclusion (13–15). The extensive effects of food insecurity underline the urgency of addressing this issue within broader public health and social equity efforts (16–18). It highlights the necessity for comprehensive strategies to ensure access to nutritious food, thus mitigating the negative health, social, and economic consequences linked to food insecurity (9, 17).

The link between educational attainment and food insecurity indicates that higher education can mitigate the risk of food insecurity by enhancing employment and income opportunities (19), emphasizing the importance of socioeconomic policies in promoting economic stability and improving access to healthy food (17, 20). However, the Minorities’ Diminished Returns theory (21) shows that racial and ethnic minorities often see less benefit from higher educational attainment due to systemic barriers such as lower-quality education and limited access to well-paying jobs (22). This discrepancy underscores the necessity for policies specifically designed to address the structural inequalities that inhibit equal benefits from educational advancements among minority groups (23).

### Aims

This study examines the disparity in the relationship between educational attainment and food insecurity among Latino and non-Latino adults via marital status and employment. It was hypothesized that marital status and employment would play some role in the observed diminished returns among Latinos. Our initial hypothesis posits a weaker inverse association between educational attainment and food insecurity among Latinos compared to non-Latinos.

## Methods

### Design and setting

The study utilized data from the 2022 National Health Interview Survey (NHIS), conducted by the Centers for Disease Control and Prevention (CDC). This survey collected comprehensive data on health status, food security, and socioeconomic status (SES) among the US population.

### National Health Interview Survey (NHIS)

The National Health Interview Survey (NHIS) is a vital source of health information in the United States. Conducted annually by the National Center for Health Statistics (NCHS), which is part of the Centers for Disease Control and Prevention (CDC), the NHIS aims to monitor the health of the U.S. population through the collection and analysis of data on a broad range of health topics. The NHIS uses a cross-sectional household interview survey design, which means it collects data from a new sample of households each year. The sample is representative of the civilian, non-institutionalized population of the U.S., excluding individuals in the military, prisons, and long-term care facilities. The survey is structured to produce reliable national estimates for various health indicators. The sampling process involves a multi-stage area probability design. This ensures that various geographic areas and demographic groups are adequately represented. Each year, about 35,000–40,000 households are selected, yielding interviews with approximately 75,000–100,000 individuals. For data collection, trained interviewers from the U.S. Census Bureau conduct face-to-face interviews using computer-assisted personal interviewing (CAPI) technology. In some cases, telephone follow-ups may be conducted. The interviews are conducted continuously throughout the year, ensuring data is collected in all seasons. The NHIS questionnaire is divided into several sections, including the Core Questionnaire which remains relatively stable year-to-year and includes basic demographic information, health conditions, health behaviors, and health care utilization. Supplemental Modules, however, change annually and focus on current public health issues, such as chronic conditions, immunizations, mental health, and more. The NHIS employs rigorous quality control procedures, including interviewer training, pre-testing of survey instruments, and continuous monitoring and evaluation of data collection processes. The NHIS collects a wide range of health-related data, including but not limited to Demographic Information (age, sex, race, ethnicity, income, education, employment status, and household composition), Health Conditions (chronic diseases such as diabetes and heart disease as well as disabilities, Health Behaviors (smoking, alcohol consumption, physical activity, diet, and sleep patterns), Health Care Utilization such as health insurance coverage, access to and use of medical care, preventive services, and hospitalizations, Mental Health (depression, anxiety, and mental well-being). While the core questionnaire provides consistent data for longitudinal analysis, the annual supplemental modules allow the NHIS to address emerging health issues and policy needs. This flexibility enables researchers to explore a wide range of health topics and trends over time.

### NHIS sample and sampling

A nationally representative sample of non-institutionalized adults aged 18–99 from across all racial/ethnic groups in the US was selected, totaling 27,648 participants.

### Measures

Age: measured in years since the last birthday, ranging from 18 to 99, and treated as a continuous variable.

Sex: coded dichotomously, with 1 indicating male.

Races included were black, native American, Asian, and white (reference group).

Independent variable: educational attainment was the independent variable, operationalized on an 11-point scale ranging from 0 (Never attended, kindergarten only) to 10 (Professional School or Doctoral degree, e.g., MD, DDS, DVM, JD, PhD, EdD), and treated as continuous.

Dependent variable: food insecurity was assessed through multiple items and defined dichotomously, with 1 indicating any level of food insecurity.

Moderator: ethnicity, categorized as non-Latino (0) or Latino (1), was considered a binary moderating variable based on self-reported ethnicity.

Marital status: in our study, marital status was categorized into two main groups: married and unmarried. Participants who were cohabiting, widowed, or had never been married were classified under the unmarried category. This classification allowed us to examine the differences in food insecurity based on marital status, capturing both the protective effects associated with marriage and the potential vulnerabilities linked to unmarried statuses.

Employment status: categorized as employed versus unemployed. The unemployed included those not employed or not in the labor market at the time of the survey.

### Statistical analyses

Data were analyzed using Stata 18.0 (StataCorp LLC, College Station, TX). Frequencies and percentages were reported in univariate analyses, while bivariate analyses examined differences between Latino and non-Latino populations across all study variables. Pearson correlation was employed to evaluate correlations between variables, with analyses stratified by ethnicity. Path modeling, a subtype of structural equation modeling (SEM), was applied to the pooled sample. This model incorporated an interaction term between educational attainment and Latino ethnicity, targeting food insecurity as the outcome variable, and considered marital status and employment as potential mediators. A positive and significant path coefficient for educational attainment with respect to employment and marital status indicated beneficial effects of education. Conversely, a negative and significant interaction between Latino ethnicity and educational attainment on these socioeconomic factors suggested that the positive impacts of education on employment and marital status are less pronounced for Latinos compared to non-Latinos. Results were presented with standardized coefficients (Beta), standard errors (SE), 95% confidence intervals (CIs), and *p*-values, with statistical significance set at *p* ≤ 0.05.

In our analysis of the National Health Interview Survey (NHIS) data, survey’s design variables, including strata, clusters, and weights were meticulously incorporated, to accurately reflect its complex sampling framework. By utilizing Stata for our statistical analysis, we ensured that the NHIS’s stratification and clustering were properly accounted for, making our results representative of the broader U.S. population. Weights were applied to adjust for both the probability of selection and non-response, ensuring that each participant’s data contributed appropriately to the analysis. For the estimation of standard errors (SE), the Jackknife method was used. This approach is a robust technique that generates reliable SE estimates by systematically re-sampling the dataset. This approach allowed us to accurately capture the uncertainty associated with our estimates, fully considering the NHIS’s complex survey design and thereby providing more precise and reliable results.

## Results

Our analysis included a total of 27,651 individuals. According to Table 1, the racial composition of the sample was 71.6% White, 12.3% Black, 1.8% American Indian or Alaska Native (AIAN), and 6.1% Asian. Among the participants, 17.2% (*n* = 4,753) identified as Latino, while 82.8% (*n* = 22,898) were non-Latino.

**Table 1:** Descriptive data, weighted proportions (*n* = 27,651).

	%	SE	95% CI	
Hispanic				
No	82.77602	0.007	81.41332	84.05837
Yes	17.22398	0.007	15.94163	18.58668
White				
No	28.3633	0.007	27.0669	29.6964
Yes	71.6367	0.007	70.3036	72.9331
Black				
No	87.6313	0.005	86.7124	88.4951
Yes	12.3687	0.005	11.5049	13.2876
Asian				
No	93.8739	0.003	93.2864	94.413
Yes	6.1261	0.003	05.587	6.7136
AIAN				
No	98.119	0.003	97.6163	98.5173
Yes	1.881	0.003	1.4827	2.3838
Gender				
Female	51.3456	0.004	50.6538	52.0368
Male	48.6544	0.004	47.9632	49.3462
Married				
No	48.45255	0.004	47.58856	49.31747
Yes	51.54745	0.004	50.68253	52.41144
Employed				
No	39.20517	0.004	38.43008	39.98573
Yes	60.79483	0.004	60.01427	61.56992
Food insecurity				
No	92.0523	0.002	91.55455	92.52316
Yes	7.9477	0.002	7.47684	8.44545
	Mean	SE	95% CI	
Age (years)	48.20	0.16	47.89	48.53
Education	5.65	0.03	5.60	5.71

AIAN, American Indian and Alaska Native.

Table 2 reveals that food insecurity was associated with being Black or Hispanic, having a lower income-to-poverty ratio, lower employment rates, and being unmarried.

**Table 2:** Bivariate correlations, unweighted (*n* = 27,651).

	Hispanic	Male	Age	Black	Asian	AIAN	Married	Employed	Food insecurity
Hispanic	1.00								
Male	0.00	1.00							
Age	−0.18*	−0.04*	1.00						
Black	−0.11*	−0.04*	−0.03*	1.00					
Asian	−0.09*	−0.01	−0.08*	−0.09*	1.00				
AIAN	0.03*	−0.01	−0.02	−0.05*	−0.03*	1.00			
Married	−0.02*	0.06*	0.08*	−0.14*	0.07*	−0.03*	1.00		
Employed	0.08*	0.11*	−0.49*	0.00	0.05*	−0.02	0.05*	1.00	
Food insecurity	0.07*	−0.03*	−0.08*	0.10*	−0.03*	0.05*	−0.12*	−0.05*	1.00

*p < 0.0 AIAN, American Indian and Alaska Native.

**Figure 1:**
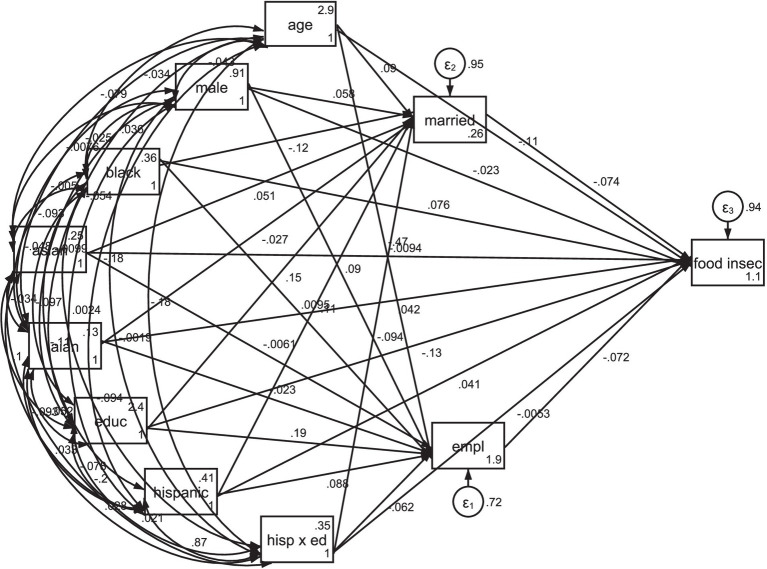
Results of path model with moderators and mediators. Educ/Ed, Educational attainment; Empl, Employment; Hisp, Hispanic/Latino; Food Insex, Food Insecurity, AIAN, American Indian and Alaska Native.

Figure 1 and Table 3 illustrate that higher educational attainment is positively associated with marital status (being married) and employment status (being employed). However, these associations were less pronounced for Latinos compared to non-Latino individuals. Additionally, being married, being employed, and having a higher level of education were all linked to reduced food insecurity. This model indicates that lower rates of employment and a lower likelihood of being married among Latinos, across all levels of education, contribute to their higher rates of food insecurity.

**Table 3:** Summary of coefficient paths with moderator and mediators.

			Beta	SE	95% CI		*p*
Age	➔	Employed	−0.47	0.00	−0.48	−0.46	< 0.001
Education x Hispanic	➔	Employed	−0.06	0.01	−0.08	−0.04	< 0.001
Black	➔	Employed	0.01	0.01	0.00	0.02	0.082
Male	➔	Employed	0.09	0.01	0.08	0.10	< 0.001
Education	➔	Employed	0.19	0.01	0.18	0.20	< 0.001
Asian	➔	Employed	−0.01	0.01	−0.02	0.00	0.254
AIAN	➔	Employed	−0.02	0.01	−0.03	−0.01	< 0.001
Hispanic	➔	Employed	0.09	0.01	0.06	0.11	< 0.001
Intercept	➔	Employed	1.90	0.02	1.85	1.95	< 0.001
							
Age	➔	Married	0.09	0.01	0.08	0.10	< 0.001
Education x Hispanic	➔	Married	−0.09	0.01	−0.12	−0.07	< 0.001
Black	➔	Married	−0.12	0.01	−0.13	−0.10	< 0.001
Male	➔	Married	0.06	0.01	0.05	0.07	< 0.001
Education	➔	Married	0.15	0.01	0.14	0.17	< 0.001
Asian	➔	Married	0.05	0.01	0.04	0.06	< 0.001
AIAN	➔	Married	−0.03	0.01	−0.04	−0.02	< 0.001
Hispanic	➔	Married	0.11	0.01	0.08	0.13	< 0.001
Intercept	➔	Married	0.26	0.03	0.21	0.32	< 0.001
							
Employed	➔	Food insecurity	−0.07	0.01	−0.09	−0.06	< 0.001
Married	➔	Food insecurity	−0.07	0.01	−0.09	−0.06	< 0.001
Age	➔	Food insecurity	−0.11	0.01	−0.12	−0.09	< 0.001
Education x Hispanic	➔	Food insecurity	−0.01	0.01	−0.03	0.02	0.686
Black	➔	Food insecurity	0.08	0.01	0.06	0.09	< 0.001
Male	➔	Food insecurity	−0.02	0.01	−0.04	−0.01	< 0.001
Education	➔	Food insecurity	−0.13	0.01	−0.15	−0.12	< 0.001
Asian	➔	Food insecurity	−0.01	0.01	−0.02	0.00	0.124
AIAN	➔	Food insecurity	0.04	0.01	0.03	0.05	< 0.001
Hispanic	➔	Food insecurity	0.04	0.01	0.01	0.07	0.003
Intercept	➔	Food insecurity	1.05	0.03	0.99	1.11	< 0.001

AIAN, American Indian and Alaska Native.

## Discussion

The primary aim of our study was to explore the link between educational attainment and food insecurity among Latino and non-Latino populations in the United States. Specifically, we investigated whether the benefits of higher educational attainment in reducing food insecurity manifest equally across Latino and non-Latino groups. The existence of disparities in the returns of education suggests that: (1) solely increasing educational levels may not suffice to bridge the gap in food insecurity for Latino individuals; (2) structural factors may underlie the reasons why education alone is insufficient; and (3) it is important to recognize that food insecurity can affect middle-class Latinos as well.

Our findings reveal that although higher education is generally associated with higher rates of marriage and employment, which are then associated with reduced food insecurity, the effects of educational attainment on marriage and employment are weaker in Latino than non-Latino individuals. Despite achieving similar educational levels, Latinos face a higher risk of food insecurity due to lower marriage rates and employment.

Previous research has established the crucial role of socioeconomic factors, including education, in enhancing economic stability and food security (24). Individuals with higher education levels are less likely to experience food insecurity, often due to improved employment opportunities and income, which facilitate access to nutritious foods (19). However, studies focusing on racially and ethnically marginalized populations indicate that the anticipated socioeconomic benefits of education do not always fully materialize, as seen in majority populations (21). This gap is central to the Minorities’ Diminished Returns theory (25, 26), which argues that structural barriers limit the effectiveness of socioeconomic advancements in improving health and well-being outcomes for marginalized groups (27).

The diminished educational returns observed among Latinos are attributed to a complex mix of societal, environmental, and structural factors (28–30). Systemic racism and discrimination, along with segregation and social stratification in education and the labor market, contribute to Latinos receiving lower-quality education and facing hurdles in securing employment commensurate with their educational attainment (31–33). Moreover, even when employed, Latinos may end up in jobs with limited financial stability and benefits, impacting their access to affordable, nutritious food (34). Social determinants of health, such as neighborhood conditions and access to healthcare, further aggravate food insecurity risks among educated Latinos (35–37).

Another explanation for our findings is the Skin-Deep Resilience theory. This theory posits that while resilient minorities may achieve success and excel in various domains, this resilience often comes at a significant cost to their physical health. Food insecurity, which has been linked to maladaptive eating behaviors, adverse cardiometabolic outcomes, and overall poorer health, may play a crucial role in this phenomenon. Even when food insecurity remains high or at baseline, it can exacerbate the risk of health problems. Researchers such as Gene Brody (38), Edith Chen (39, 40), and David Williams and Natalie Slopen (41) have extensively published on how the “Skin Deep Resilience” theory explains the linkages between upward mobility and poorer health outcomes, particularly within Black populations. Their work highlights that the stress and challenges associated with striving for upward mobility can lead to long-term negative health effects, despite achieving success in other areas of life. This perspective underscores the complex interplay between socio-economic advancement and health disparities, emphasizing the need for policies that address both economic and health inequalities comprehensively.

Mezuk and Jackson, along with their colleagues, developed the Environmental Affordance Model to elucidate the complex interactions between individual behaviors and environmental contexts, particularly concerning health disparities. This model emphasizes how environmental factors such as physical resources (e.g., availability of parks or grocery stores), social support networks, and economic opportunities shape individual health behaviors and outcomes. It posits that individual behaviors cannot be fully understood without considering the broader environmental context, including neighborhood characteristics, social norms, and institutional policies. By identifying the opportunities and constraints within an environment, the model highlights how unequal distribution of resources contributes to health inequities among different socioeconomic and racial/ethnic groups. This framework not only aids researchers in exploring the multi-level determinants of health but also guides the development of targeted public health interventions and policies to promote health equity across diverse populations.

Addressing these multifaceted drivers requires multisectoral policy responses that range from education and the labor market to civil engineering and community empowerment (21). Discrimination in hiring practices, wage disparities, occupational segregation, and the devaluation of credentials obtained by minorities are key issues that perpetuate food insecurity among educated Latinos (16). Systemic and structural inequalities may hinder educational attainment from acting as a protective factor against food insecurity for racialized populations (42, 43). A true solution needs to be community-engaged (44, 45).

### Limitations

This study offers valuable insights into the relationship between educational attainment and food insecurity among Latino and non-Latino populations. However, it faces several limitations. Its cross-sectional design precludes drawing causal inferences or tracking changes over time, limiting our understanding of the long-term effects of education on food insecurity. The analysis, confined to data from 2022, provides a temporal snapshot that may not capture evolving trends in different socioeconomic or political climates. Furthermore, the simplistic measure of educational attainment may overlook nuances such as the quality of education, field of study, and the reputation of educational institutions. While focusing on Latino and non-Latino populations, our findings may not extend to other ethnic groups with distinct experiences of systemic discrimination or differing education-food insecurity relationships. Not incorporating wealth and other socioeconomic status (SES) indicators beyond education, employment, and income represents a significant gap. This study did not include psychosocial measures such as depressive symptoms, anhedonia, adverse childhood experiences that could be correlated with ethnicity, SES, and food insecurity. Moreover, the study did not explore the experiences of other marginalized groups or the impact of state-level policies on food insecurity, which could vary widely across regions.

### Implications and future research

Our findings emphasize the need for targeted policy and practice interventions to overcome the specific challenges Latino populations face in leveraging educational attainment for food security. Improving education quality for Latino and other marginalized communities, eliminating job market discrimination, and supporting Latinos in their transition from education to employment are crucial. Additionally, addressing broader socioeconomic disparities and enhancing nutritious food access through community initiatives and federal programs could alleviate food insecurity impacts. Future research should adopt a longitudinal approach to better understand the dynamic relationship between educational attainment and food insecurity, including the long-term impacts of education on economic stability and health outcomes. Investigating policy solutions’ efficacy in reducing food insecurity among minority populations and expanding research scope to include other ethnic groups and regional variations could offer new insights. Moreover, exploring the heterogeneity within the Latino population regarding origin, migration history, and socioeconomic diversity would deepen our understanding of how education impacts food security. Future studies should also examine other factors, such as schooling quality and labor market barriers, to provide a comprehensive view of the obstacles to achieving food security among minority populations with higher education levels.

## Conclusion

In conclusion, our study underscores the importance of targeted interventions that address the root causes of diminished educational returns in Latino and other marginalized communities. Ensuring that the benefits of education reach all individuals, regardless of racial or ethnic background, requires a comprehensive strategy that integrates educational, economic, and social policies.

## Data Availability

Publicly available datasets were analyzed in this study. This data can be found here: https://www.cdc.gov/nchs/nhis/data-questionnaires-documentation.htm.

## Author disclaimer

The opinions and conclusions expressed are solely those of the author(s) and do not represent the opinions or policy of SSA or any agency of the Federal Government. Neither the United States Government nor any agency thereof, nor any of their employees, makes any warranty, express or implied, or assumes any legal liability or responsibility for the accuracy, completeness, or usefulness of the contents of this report. Reference herein to any specific commercial product, process or service by trade name, trademark, manufacturer, or otherwise does not necessarily constitute or imply endorsement, recommendation or favoring by the United States Government or any agency thereof.
